# The impact of artificial intelligence (AI) in academic writing and publication: Iranian Journal of Basic Medical Sciences (IJBMS) policy

**DOI:** 10.22038/ijbms.2025.25229

**Published:** 2025

**Authors:** Leila Arabi, Ali Roohbakhsh, Bizhan Malaekeh-Nikouei, Bibi Sedigheh Fazly Bazzaz

**Affiliations:** 1 Nanotechnology Research Center, Pharmaceutical Technology Institute, Mashhad University of Medical Sciences, Mashhad, Iran (Assistant Editor); 2 Pharmaceutical Research Center, Pharmaceutical Technology Institute, Mashhad University of Medical Sciences, Mashhad, Iran (Assistant Editor); 3 Biotechnology Research Center, Pharmaceutical Technology Institute, Mashhad University of Medical Sciences, Mashhad, Iran (Editor–in–Chief)

**Keywords:** Anti-oxidant enzymes, Apoptosis, Hippocampus, PGC1α, Type 2 diabetes

## Abstract

**Objective(s):**

This study aimed to determine the effect of 8-week high-intensity interval training (HIIT) on oxidative stress and apoptosis in the hippocampus of male rats with type 2 diabetes (T2D). The study focused on examining the role of proliferator-activated receptor gamma co-activator 1α (PGC1α)/Kelch-like ECH-associated protein Keap1/nuclear factor erythroid 2-related factor 2 (Nrf2) signaling pathway.

**Materials and Methods:**

Twenty-eight 8-week-old Wistar rats were randomly assigned to one of four groups (n=7): control (Con), type 2 diabetes (T2D), exercise (Ex), and exercise + type 2 diabetes (Ex+T2D). The Ex and Ex+T2D groups completed an 8-week exercise program consisting of 80-100% Vmax and 4–10 intervals. The homeostasis model assessment of insulin resistance (HOMA-IR) index was used to assess insulin resistance. The levels of Bcl2, BAX, musculoaponeurotic fibrosarcoma (Maf), Nrf2, Keap1, and PGC1α in the hippocampus were assessed using the western blot method. Additionally, the levels of antioxidant enzymes in the hippocampus were measured using ELISA.

**Results:**

The findings indicated that the T2D group had lower levels of antioxidant enzymes, Maf, Bcl2, PGC1α, and Nrf2, and higher levels of BAX and Keap1 in the hippocampus. Conversely, the HIIT group exhibited increased levels of antioxidant enzymes, Maf, Bcl2, Nrf2, and PGC1α, along with decreased levels of BAX and Keap1 in the hippocampus.

**Conclusion:**

The study demonstrated that 8-week HIIT was effective in reducing hippocampal apoptosis and oxidative stress induced by T2D by activating the PGC1α-Keap1-Nrf2 signaling pathway. The metabolic changes induced by exercise may lead to an increase in PGC1 expression, which is the primary stimulator of the Keap1-Nrf2 signaling pathway.

## Introduction

The rise of artificial intelligence (AI) has attracted significant interest across various areas including academic publishing. As AI technologies such as machine learning and large language models (LLMs) become increasingly sophisticated, they promise to revolutionize the way scientific research is written, reviewed, and even published. While AI’s potential for enhancing efficiency, accuracy, and accessibility is widely welcomed, its integration into academia also raises critical questions about authenticity, ethical concerns, and the future of academic work. In this editorial we aim to explore the significance, hopes, and hypes surrounding AI’s role in academic writing and scientific publishing.

***The significance of AI in academic writing***

AI’s role in academic writing has already proven valuable in a variety of ways, from drafting research papers to improving writing clarity and coherence. Researchers now have access to writing assistants that can suggest improvements in grammar, style, and structure. Moreover, AI-based systems are capable of summarizing vast amounts of data, conducting preliminary research, and even suggesting potential areas for exploration (1).

Notably, the significance of AI is not just limited to writing assistance, AI systems are already being used to facilitate the peer-review process and assist in data analysis. For instance, automated tools can screen manuscripts for plagiarism, check for errors, and even evaluate the research quality, enabling editors to make quicker, more informed decisions (2). 

***AI hopes in academic publishing***

The promises of AI in academic publishing are substantial. Many hope that AI will make easier and broader access to academic knowledge by making the publication process more efficient, transparent, and accessible to researchers worldwide. For example, AI systems could help underrepresented researchers in developing countries access advanced tools for publishing their work and enabling global scientific collaboration (3).

***The hypes and challenges of AI in academic publishing***

Despite the excitement surrounding AI’s potential, there is also a great deal of hype and limitations that must be addressed. For instance, AI models often rely on large datasets that can sometimes contain biases. These biases may be unconsciously reinforced in academic publications, leading to biased interpretations of research. Another concern is the loss of academic integrity. AI can be used to generate large volumes of text, but the it is important that to what extent can we trust that the content produced by AI is original, accurate, and reflective of human thought? The AI-generated content raises questions about authorship, plagiarism, and the value of human insight in research (1). 

Therefore, there is an ongoing debate about the ethical implications of AI in academic publishing. If AI systems are tasked with conducting peer reviews or suggesting edits to manuscripts, who is responsible for the final manuscript? Should AI allowed to assess research quality? The answers to these questions can have profound consequences for the development of academic publications. 

***Iranian Journal of Basic Medical Sciences (IJBMS) policy on artificial intelligence*** ***issue***

AI in academic writing and publishing presents both opportunities and challenges. However, the application of AI must be approached with caution. IJBMS guideline and policy is in agreement with the Committee on Publication Ethics (COPE) position statement on the use of AI at any step of manuscript preparation. We have clearly indicated that “Authors are fully responsible for the content of their manuscript, even those parts produced by an AI tool, and are thus liable for any breach of publication ethics” (4).

We do our best to address ethical concerns and manage the hype surrounding AI, to control its power for the greater good of our audience to enhance scientific publishing integrity. At the end, we wish all the academic communities around the world a HAPPY NEW YEAR.

## References

[1] Carobene A, Padoan A, Cabitza F, Banfi G, Plebani M (2024). Rising adoption of artificial intelligence in scientific publishing: Evaluating the role, risks, and ethical implications in paper drafting and review process. Clin Chem Lab Med.

[2] Livberber T, Ayvaz S (2023). The impact of artificial intelligence in academia: Views of Turkish academics on ChatGPT. Heliyon.

[3] Butson R, Spronken-Smith R (2024). AI and its implications for research in higher education: A critical dialogue. High Educ Res Dev.

[4] Arabi L, Roohbakhsh A, Malaekeh-Nikouei B, Fazly Bazzaz BS (2024). Joining COPE: Opportunities and benefits for the Iranian Journal of Basic Medical Sciences. Iran J Basic Medl Sci.

